# Supervised Estimation of Granger-Based Causality between Time Series

**DOI:** 10.3389/fninf.2017.00068

**Published:** 2017-11-29

**Authors:** Danilo Benozzo, Emanuele Olivetti, Paolo Avesani

**Affiliations:** ^1^NeuroInformatics Laboratory, Bruno Kessler Foundation, University of Trento, Trento, Italy; ^2^Information Engineering and Computer Science Department (DISI), University of Trento, Trento, Italy; ^3^Center for Mind and Brain Sciences (CIMeC), University of Trento, Trento, Italy

**Keywords:** causal inference, brain effective connectivity, Granger causality, machine learning, Geweke measure in time, causal interaction classification

## Abstract

Brain effective connectivity aims to detect causal interactions between distinct brain units and it is typically studied through the analysis of direct measurements of the neural activity, e.g., magneto/electroencephalography (M/EEG) signals. The literature on methods for causal inference is vast. It includes model-based methods in which a generative model of the data is assumed and model-free methods that directly infer causality from the probability distribution of the underlying stochastic process. Here, we firstly focus on the model-based methods developed from the Granger criterion of causality, which assumes the autoregressive model of the data. Secondly, we introduce a new perspective, that looks at the problem in a way that is typical of the machine learning literature. Then, we formulate the problem of causality detection as a supervised learning task, by proposing a classification-based approach. A classifier is trained to identify causal interactions between time series for the chosen model and by means of a proposed feature space. In this paper, we are interested in comparing this classification-based approach with the standard Geweke measure of causality in the time domain, through simulation study. Thus, we customized our approach to the case of a MAR model and designed a feature space which contains causality measures based on the idea of precedence and predictability in time. Two variations of the supervised method are proposed and compared to a standard Granger causal analysis method. The results of the simulations show that the supervised method outperforms the standard approach, in particular it is more robust to noise. As evidence of the efficacy of the proposed method, we report the details of our submission to the causality detection competition of Biomag2014, where the proposed method reached the 2nd place. Moreover, as empirical application, we applied the supervised approach on a dataset of neural recordings of rats obtaining an important reduction in the false positive rate.

## 1. Introduction

A main part of neuroscience research is concerned with brain connectivity and aims to investigate the pattern of interactions between distinct units within the brain (Horwitz, 2003). The concept of brain units is strongly related to the level of the adopted scale. Thus, brain connectivity can be studied from the microscopic level of single synaptic connections to the macroscopic level of brain regions. Moreover, depending on the type of interactions of interest, brain connectivity is divided into *structural, functional*, and *effective* connectivity. In the first case the connectivity patterns are referred to the anatomical links i.e., the neural pathways. In the second case, to the statistical dependencies between brain activity in different units. In the last case, the connectivity patterns are referred to the causal interactions between them (Sakkalis, 2011). In particular, effective connectivity provides information about the direct influence that one or more units exert over another and aims to establish causal interactions among them (Friston, 2011).

Electrophysiological signals are among the most suitable ones for studying effective connectivity. First, because they directly measure neuronal activity, even though at an aggregated level. Second, because their temporal resolution is compatible with the processing time at the neuronal level, that is in the order of milliseconds (Schoffelen and Gross, 2009). These signals can be measured with invasive or non-invasive methods. Invasive methods allow a high quality and spatially precise acquisition, by implanting electrodes on the brain. On the other side, non-invasive techniques such as magneto- and electro-encephalography (M/EEG) are widely used because of the high sampling frequency and, by means of source reconstruction techniques, they provide increased signal-to-noise ratio and spatial resolution (Brookes et al., 2012).

The interest in studying causal interactions from neuroimaging data is not only limited to effective connectivity but it has a more general scope. The original definition of effective connectivity provided in Friston (2011), refers to the directed influences that neuronal populations in one brain area exert on those in another one. Thus an estimator of effective connectivity should consider the physiological structure and dynamics of the system (Friston et al., 2014). This constraint is particularly demanding since it means modeling the underlying physical processes. To overcome such issue, a relaxed version of effective connectivity was introduced in Bressler and Seth (2011) under the name of *causal connectivity*. Causal connectivity refers to a causality measure that infers the causality structure without requiring it to be representative of the underlying neuronal network. The term *causality analysis* is commonly used when studying the direct interactions among brain signals. As highlighted in Chicharro and Ledberg (2012), a causality analysis may have different meanings. Its purpose could be to infer the existence of a direct causal connection, thus the estimate of the so-called causal structure or (binary) causal graph (Eichler, 2005). A different goal is to study the mechanism underlying a causal connection. This means focusing on how a causal connection is physiologically implemented. And a third question concerns the quantification of the interaction, thus it requires both an appropriate modeling of the dynamics and a clear understanding of what the causal effect actually means, see Schelter et al. (2009).

In this work, we focus on the problem of inferring the binary causal graph from a given set of time series. This means that our purpose is to establish the existence of causal interactions without necessarily considering the underlying mechanism and quantification issues.

### 1.1. Approaches for causal inference

Different frameworks have been proposed to infer causality, e.g., Potential Outcomes (Holland, 1986), Granger Causality (Granger, 1969), Dynamic Causal Modeling (Friston et al., 2003), Causal Bayesian Networks (Pearl, 2000), and Structural Equation Models (Spirtes et al., 2001). These frameworks differ in many aspects and a main one is the assumption on the input data, which can be observational or interventional. Here, we focus on the case of causal inference from purely observational data, in particular time series.

**Figure 1:**
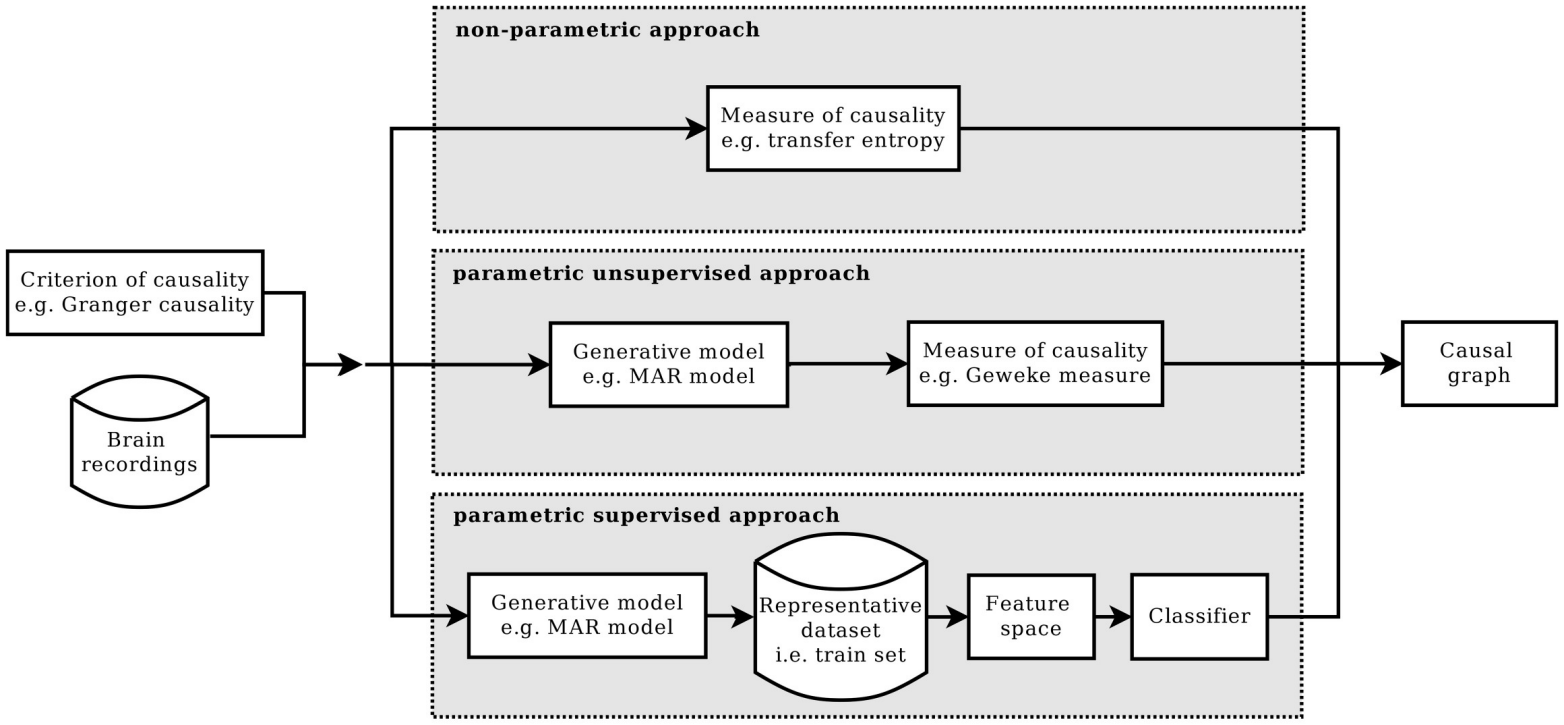
Given a criterion of causality, the estimation of causality structure can be implemented in three different ways: the standard non-parametric approach (top), the parametric one (mid), and the proposed parametric supervised one (bottom).

Commonly, a method of causal inference is based on a specific *causality criterion* from which a measure of causality is derived (Chicharro, 2014). A criterion of causality defines which condition has to be satisfied in order to establish that two processes are causally interacting, or not. Given a certain criterion and according to how it is formulated, different measure of causality can be developed. There are cases in which the measure is defined by assuming a model for the underlying process of data generation, the so-called parametric formulations of the criterion. Or in case of a model-free approach, the formulation is said to be non-parametric. Figure 1 summarizes the main blocks of these two approaches and introduces the main blocks of the alternative approach that is proposed in this paper, which is called the *parametric supervised approach*. The figure is horizontally divided in three parts, one for each approach. They all start by requiring a criterion of causality and a multivariate time series, as input dataset. And they all end with an estimate of the casual graph of the input dataset.

In the parametric approach, a criterion of causality is chosen and then, according to it, a model of the generative process is assumed and a measure for causality is defined. Commonly, the computation of the causality measure requires the identification of the model, which, in general, is not trivial (Valdes-Sosa et al., 2011). Moreover, to obtain the causal graph from the computed measures, the significance of the non-zero values needs to be tested. This can be done, for example, by means of bootstrap techniques, or by knowing the actual distribution under the null hypothesis.

In the non-parametric approach, given a criterion of causality, its definition of causal interaction is formulated in terms of equations between probability distributions. Afterwards, a metric is adopted in the information-theoretic framework in order to test whether the equality holds (Solo, 2008; Vicente et al., 2011).

Differently from the parametric and non-parametric approach, here we propose a novel direction to attack the problem of detecting causality, which we call *supervised parametric* approach. The supervised approach is based on machine learning techniques and, specifically, on learning from examples. Each example comprise a multivariate time series together with their true causal structure. The idea of proposing causal inference as a learning theory problem is not new, especially in the area of observational data causality (Schölkopf et al., 2013; Mooij et al., 2016). One of the first examples is (Lopez-Paz et al., 2015a,b), where the authors adopted a supervised approach for bivariate causal inference with the use of kernel mean embeddings for feature mapping. Here, the same idea of a supervised detection of causal interactions is used but with a different implementation. Moreover, we specifically target the context of time series analysis. In our variant, the model is not used to derive a measure but to generate a dataset of multivariate time series together with their actual causal graphs. The purpose of this dataset is to be used as *training set* for a classification algorithm, aimed to predict the causal graph of future multivariate time series.

A consequence of the proposed approach is that we need to build a feature space in which to represent the dataset, such that the specific aspects of the chosen causality criterion are represented. Moreover, it is interesting to notice that model and feature space do not need to derive from the same causality criterion. This means that the proposed supervised approach allows to disentangle the mechanism of data generation from the criterion used to describe the causal structure.

In this work, the proposed supervised parametric approach is compared with the standard parametric formulation. For this reason, we refer to the standard parametric approach as to the *unsupervised parametric* one. In the context of the Granger criterion of causality (Granger, 1969), we conduct the comparison through a simulation study. Granger causality is the most adopted criterion for causal inference in brain recordings (Seth et al., 2015) and it is based on the assumptions of precedence and predictability of the cause with respect to its effect. Precedence means that a cause has to temporally precede its effect. Predictability is referred to the conditional dependence that exists between the past of the causes and the future of the effect, conditioned on the past of the effect itself.

### 1.2. Causality measures based on the granger criterion

In the following, we provide a brief summary of the most important measures of causality that have been developed from the Granger criterion, both for the non-parametric and parametric cases.

For the non-parametric approach, a widespread causality measure is transfer entropy, which compares the probability distributions between the candidate effect and the past of the candidate cause, under the hypothesis of independence (Schreiber, 2000; Amblard and Michel, 2010, 2012). Specifically, transfer entropy computes the Kullback-Leibler divergence between the probability distribution of the candidate effect conditioned on it own past and the same effect conditioned also the past of the candidate cause. By definition, this measure is non-negative and zero only when the two distributions are equal. Moreover, the fact that KL-divergence does not consider any specific statistical moment of a given order, is particularly suited for detecting non-linear interactions. Beyond transfer entropy, other non-parametric measures have been proposed (Ancona et al., 2004), such as the measure based on Fisher information.

The parametric representation of the Granger criterion assumes a linear autoregressive model of the process. This assumption refers to how time series are interacting with each other, without explicitly modeling the physical mechanism of generation. The autoregressive representation has led to different formulations of measures of causal interaction. The temporal formulation tests the presence of causality by comparing the residual variances of the effect in which the candidate cause is initially excluded vs. when it is included, during model identification. The causal measure is defined as the natural logarithm of the ratio of the residual variances, that we refer to as the *Geweke measure in time domain*. A meaningful reduction of the residual variance when the candidate cause is included in model identification means a better model for the effect. In such case, the time series evaluated as possible cause is said to *Granger cause* the time series evaluated as effect (Bastos and Schoffelen, 2015). We will use this measure as baseline estimate of causality in the upcoming experiments. In the literature, the *Geweke measure* is also known as the *Granger index*, here we adopt the choice of Chicharro (2014) to distinguish between the criterion and the measure and so to refer to the criterion as the *Granger criterion* and to the measure as the *Geweke measure*. It has been proven that this measure of causality is a test of Granger causality on the first moment statistic of the underlying probability distributions (Granger, 1980), since it is based on the linear assumption of the process. This is in contrast with transfer entropy where, by definition, the whole probability distribution of the processes is considered (Barnett et al., 2009).

The autoregressive parametric formulation of the Granger criterion was also implemented in the spectral domain. It was introduced in Geweke (1982) and named *Geweke spectral measure* of Granger causality. In the bivariate case, the Geweke spectral measure from *x* and *y* at the frequency ω, is defined as the natural logarithm of the ratio of the power spectrum of *y* computed considering the possible contribution of *x* and the power spectrum of *y* computed alone, in both cases evaluated at ω. It is interpreted as the portion of the power spectrum associated with the residuals that do not take into account the presence of *y* (Chicharro, 2011). The Geweke spectral measure does not have its equivalent formulation in the information-theoretic framework. As shown in Chicharro (2011), the lack of a temporal separation between the past and the future of the involved processes is what defines a spectral formulation of a parametric formulation. Differently, in the non-parametric formulation, a spectral measure is not available, because no way to avoid temporal separation has been proposed yet.

Other examples of causal measures developed in the spectral domain are the Partial Directed Coherence (PDC) (Baccalá and Sameshima, 2001) and the Direct Transfer Function (DTF) (Kaminski and Blinowska, 1991). Both were initially developed under the assumption of identity matrix as covariance matrix of the innovation process and then generalized in Takahashi D. Y. et al. (2010b), where they are named the information PDC (iPDC) and the information DTF (iDTF). Both are defined as a coherence measure between two processes thus they have an interpretation in term of mutual information rate. Moreover, both are measures to test for Granger causality, but only in the case of DTF, a direct connection between the bivariate Geweke spectral measure and the bivariate iDTF exists. iPDC assumes an autoregressive model for the process while iDTF starts with the moving average representation of the autoregressive model.

In the neuroscience domain, the multivariate extension of the causality measures introduced so far has great importance (Pereda et al., 2005). In the case of the bivariate iPDC and iDTF, the multivariate extension are straightforward (Takahashi D. et al., 2010a). Also the Geweke measure in time domain has a direct multivariate extension from the bivariate case, by conditioning on the processes that are not included in the pair (Barrett et al., 2010). Less immediate is the extension of the spectral representation: for a detailed explanation see Geweke (1984).

### 1.3. Proposal

The aim of this work is to investigate the proposed supervised formulation by adopting a parametric model of the Granger criterion of causality. We propose a simulation study in the context of the autoregressive model, specifically in the time domain. With these ingredients, it is possible to have a fair comparison against the standard conditional Geweke measure in time domain. Across the experiments, we compare the proposed method against a standard Granger causal analysis (GCA) method (Barnett and Seth, 2014). In particular, our interest is in facing the problem of high false positive rate that is typical for the Geweke measure when applied on noisy data. Moreover, we aim to overcome the fact that most of the approaches based on the Granger criterion, are also pairwise-based. And so they do not consider the multivariate nature of the signals. The way used to face these problems includes the supervised framework and a definition of a feature space that takes into account the multivariate aspect.

The proposed approach is analyzed in a series of experiments that are grouped in two parts. What differs between them is the generative process used for the training and for the testing/prediction phase. In the first group, the model is the same for the training and the testing phases. The first group is meant to evaluate the proposed approach under the three main aspects of the method: the generative model, the feature space and the classification task. In the second group, the generative model differs between training and testing sets. This case is quite common in practical cases, because the recorded signals may not fully respect the assumptions of the generative model assumed for the analysis.

In addition, we report the details of the solution computed with the supervised method that we submitted to the Biomag2014 Causality Challenge (Causal2014)b^1^, which reached the second place of the ranking (Benozzo et al., 2016). Such competition adopted an autoregressive model as generative process to simulate brain signals. The model generated a three-dimensional multivariate time series, given a randomly generated causal graph^2^. The competition distributed a large set of these multivariate time series and the task was to reconstruct their causal graphs.

In the second part of the experiments, we introduced a mismatch between the generative process of the training phase and the process of the prediction phase. The purpose of studying such situation is to assess how strong is the bias of the generative model, i.e., the one used to create the training set, when predicting data coming from a (partly) different process. Two different cases are analyzed in the second part: one with simulated datasets and the second with neural recordings from rats.

## 2. Materials

In this section, we describe the multivariate autoregressive model (MAR) used in our simulations and then the neural recordings used for testing the proposed method in a real setting.

### 2.1. The MAR model

The final output of the MAR model is the multivariate time series **X** = {*X*(*t*), *t* = 0, 1, …, *N* − 1}, *X*(*t*) ∈ ℝ^*M*×1^ that is defined as the linear combination of two *M*-dimensional multivariate time series **X_s_** and **X_n_**

(1) $$X=(1-\gamma )X_{s}+\gamma X_{n}$$

**X_s_** carries the causal information, **X_n_** represents the noise corruption and γ ∈ [0, 1] tunes the signal-to-noise ratio. The choice of this formulation of the MAR model, with additive noise included, is motivated by the facts that Granger metrics are strongly affected by both uncorrelated and linearly mixed additive noise (Vinck et al., 2015; Haufe and Ewald, 2016) and because it was also adopted in the Causal2014 competition. Each time point of **X_s_** and **X_n_** is computed by following the MAR model

(2) $$\begin{array}{l} X_{s}(t)=\sum_{\tau =1}^{p}A_{s}^{(\tau )⊤}X_{s}(t-\tau )+\varepsilon_{s}(t)\\ X_{n}(t)=\sum_{\tau =1}^{p}A_{n}^{(\tau )⊤}X_{n}(t-\tau )+\varepsilon_{n}(t) \end{array}$$

where *p* is the order of the MAR model and represents the maximal time lag, ε_*s*_(*t*) and ε_*n*_(*t*) are realizations from a *M*-dimensional standard normal distribution and $A_{s}^{\left(\tau \right)},A_{n}^{\left(\tau \right)}\in {\mathbb{R}}^{M\times M},\tau =1,\ldots ,p$, are the coefficient matrices modeling the influence of the signal values at time *t* − τ on the current signal values, i.e., at time *t*. The coefficient matrices $A_{s}^{\left(\tau \right)}$ are involved in the process of causal-informative data generation. They are computed by multiplying the non-zero elements of the *M* × *M* binary matrix *A* with uniformly distributed random numbers. In essence, *A* is called causal configuration matrix and represents the causal graph that leads the MAR model. Specifically *A*_*ij*_ = 1 means signal *i* causes the signal *j*. On the other hand, coefficient matrices $A_{n}^{\left(\tau \right)}$ lead the noisy part of the signals and they are obtained by randomly generating *p* diagonal matrices. After that, if both sets of matrices $A_{s}^{\left(\tau \right)}$ and $A_{n}^{\left(\tau \right)}$ fulfill the stationarity condition, each time point of *X*_*s*_ and *X*_*n*_ is generated by Equation (2).

### 2.2. Neural recording dataset

The neural recording data that have been used for the real application experiment, belong to the *hc-3 dataset* (Mizuseki et al., 2009, 2013). The dataset and related details on the acquisition are available online at https://crcns.org/data-sets/hc/hc-3. Neural time series were recorded from rats while they were performing multiple behavioral tasks. We only used local field potentials from session eco013.156 of three specific shank probes, i.e., the ones associated to the Cornu Ammonis (CA1) and the entorhinal cortex (EC3 and EC5). Each shank has eight recoding sites. Signals were low pass filtered at 140 Hz, down-sampled at 600 Hz and epoched into non-overlapping segments of 5 s duration. Moreover, we averaged across recording sites in each shank. Our final dataset contains 102 trials each of 3 time series with 5 s length associated to the three brain areas (CA1, EC3, and EC5). In order to quantify the accuracy of the evaluated methods, the true causal graph was defined by assuming the following chain of interactions: EC3→CA1→EC5, as in van Strien et al. (2009).

## 3. Methods

In this paper, we propose a parametric supervised approach to the problem of causal inference. The idea is to define the causal inference in a supervised machine learning framework, in which a classifier learns how to discriminate among a set of predefined classes, i.e., causal configurations, though a training phase. The approach is *parametric* because a model of the generative process is assumed and used to generate examples for the training phase. In details, there are two main ingredients to handle the problem in a parametric supervised way: the first is a model for the stochastic process underling the time series and the second is a feature space able to capture the causal relationships of a given set of time series. The choice of the model is a step in common with all other parametric criteria for causal inference. The difference is that, in our case, the model is used for the generation of the training set instead of the formulation of a measure of causality. In order to compare the supervised framework with the Geweke measure of causality in time domain, we instantiated our method with the MAR model. Moreover, we designed a feature space based on the idea of predictability and precedence in time, as in the Geweke measure^3^. In the following we report all the details of this procedure.

### 3.1. Data generation and causal configuration

The training dataset, that is class-*labeled* and denoted as **L**, is generated considering the total number of causal configuration matrices *A* that can be produced by a given number of time series. In a general setting, each trial **X** is composed by *M* time series and the final goal of causal inference is to estimate its *M* × *M* configuration matrix *A*. Thus, there are *M*(*M* − 1) free binary parameters and 2^*M*(*M*−1)^ possible causal configuration matrices^4^. Considering that **L** must be representative of the entire population of configurations, it will be generated so that multiple trials are included for each possible causal graph.

### 3.2. Classification schema: MBC and CBC

Here we describe two versions of the parametric supervised method, they differ in the definition of the class label. In the first, the entire causal configuration matrix *A* is considered the class label of the trial. This choice implies that one classifier is trained to discriminate among 2^*M*(*M*−1)^ classes. We will refer at this solution as the *matrix-based* classification (MBC) method. In the second version of the parametric supervised method, each cell of the configuration matrix is analyzed independently from other cells. Since each cell can be only 0 or 1, then the whole problem of predicting the causal configuration is transformed into *M*(*M* − 1) binary classifications problems, one for each cell. We call this approach the *cell-based* classification (CBC).

### 3.3. Definition of the feature space

The feature space is defined on the same assumptions done in the case of the autoregressive implementation of Granger causality. Thus, each trial is mapped into a vector of measures, that quantify the ability to predict one time series at a given time point, i.e., the *effect*, from the past of each possible subset of the *M* time series in the trial, i.e., the possible *causes*. We call the pair, made by causes and effect, *causality scenario*. In other words, chosen one of the *M* time series as the effect in the causality scenario, the related possible causes are all the subsets that can be formed from the whole set of time series. For *M* time series, the number of scenarios is ∑i=1MMiM=(2M-1)M, by using the binomial theorem. In Table 1, we report the causality scenarios in the case of *M* = 3. Thus, the possible causality scenarios are 7 for each *x*_*i*_(*t*), *i* = 0, 1, 2, i.e., time series that defines a trial, so 21 causality scenarios in total.

**Table 1:** For each effect *x*_*i*_(*t*) and *M* = 3, we report the seven possible causality scenarios.

	**Causes**	**Effect**
1	*x*_0_(*t*)	*x*_*i*_(*t*)
2	*x*_1_(*t*)	*x*_*i*_(*t*)
3	*x*_2_(*t*)	*x*_*i*_(*t*)
4	*x*_0_(*t*), *x*_1_(*t*)	*x*_*i*_(*t*)
5	*x*_0_(*t*), *x*_2_(*t*)	*x*_*i*_(*t*)
6	*x*_1_(*t*), *x*_2_(*t*)	*x*_*i*_(*t*)
7	*x*_0_(*t*), *x*_1_(*t*), *x*_2_(*t*)	*x*_*i*_(*t*)

**Figure 2:**
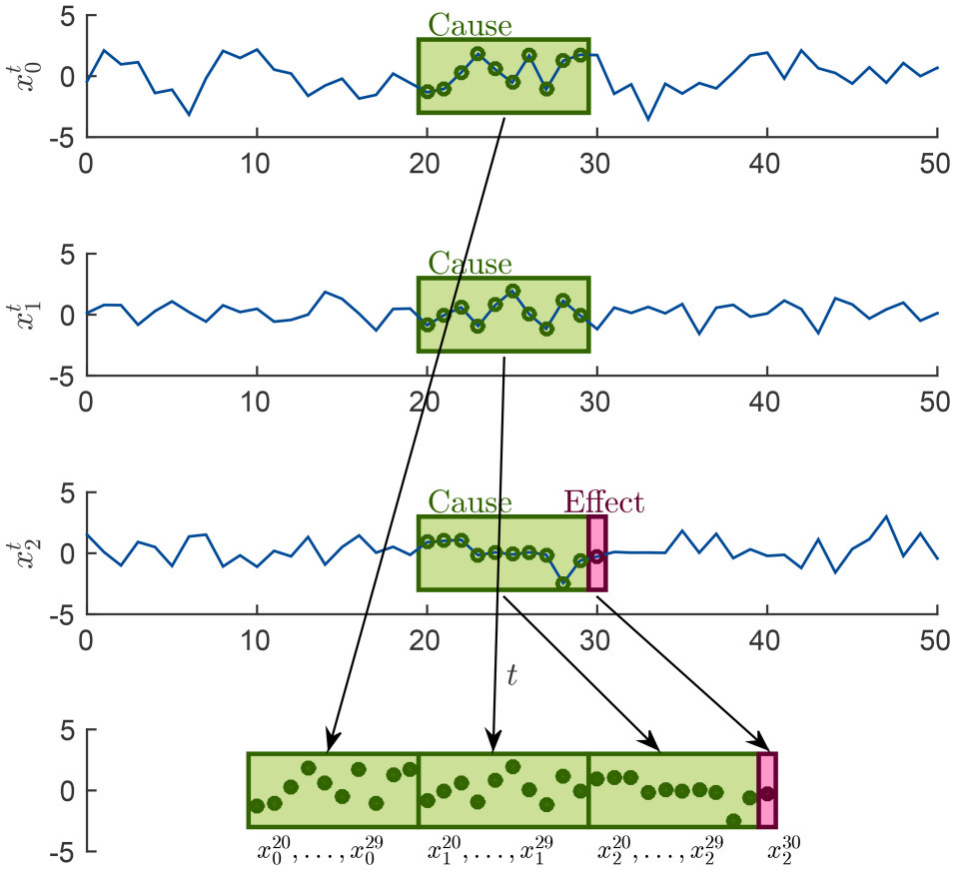
Example of how the sample associated at the time point *t* = 30 is built in order to form the input of the last regression problem in Table 2, for the case *i* = 2 and *p* = 10.

For each causality scenario, a plain linear regression problem is built by selecting, as dependent variable, the time points from the signal in the *effect* column. Each of these dependent variables has a regressor vector composed by the *p* previous time points selected from the signals in the *causes* column, where *p* is the order of the MAR model, see section 2.1. Table 2 shows how the regression problems are defined when *M* = 3, by specifying from which time series and time points, regressors and dependent variables are extracted. In the following, in order to simplify the notation, we will use $x_{i}^{t}$ instead of *x*_*i*_(*t*), *i* = 0, 1, 2 and *t* ∈ **T**, **T** ⊆ {*p*, …, *N* − 1}. Figure 2 explains how, for the specific time point *t* = 30 and for *p* = 10, the input of the regression problem is built for the last causality scenario (7) of Table 2 and *i* = 2: {*x*_0_, *x*_1_, *x*_2_} → *x*_2_. Lastly, the regression problem of each causality scenario is scored, by common metrics like the means squared error. Such scores are used as features in the feature space representation of the training set **L**.

**Table 2:** Description of how the 21 linear regression problems are defined for each trial.

**Regressors (causes)**	**Dependent variable (effect)**
$\left[x_{0}^{t-p},\ldots ,x_{0}^{t-1}\right]$	$x_{i}^{t}$
$\left[x_{1}^{t-p},\ldots ,x_{1}^{t-1}\right]$	$x_{i}^{t}$
$\left[x_{2}^{t-p},\ldots ,x_{2}^{t-1}\right]$	$x_{i}^{t}$
$\left[x_{0}^{t-p},\ldots ,x_{0}^{t-1},x_{1}^{t-p},\ldots ,x_{1}^{t-1}\right]$	$x_{i}^{t}$
$\left[x_{0}^{t-p},\ldots ,x_{0}^{t-1},x_{2}^{t-p},\ldots ,x_{2}^{t-1}\right]$	$x_{i}^{t}$
$\left[x_{1}^{t-p},\ldots ,x_{1}^{t-1},x_{2}^{t-p},\ldots ,x_{2}^{t-1}\right]$	$x_{i}^{t}$
$\left[x_{0}^{t-p},\ldots ,x_{0}^{t-1},x_{1}^{t-p},\ldots ,x_{1}^{t-1},x_{2}^{t-p},\ldots ,x_{2}^{t-1}\right]$	$x_{i}^{t}$

$x_{i}^{t}$, *i = 0, 1, 2 and t ∈ **T**, **T** ⊆ {p, …, N − 1}, are the three time series of a trial*.

### 3.4. Relationship with the geweke measure

As summarized in Table 1, the feature space is defined by exploiting all possible causality scenarios among a set of *M* time series. Differently, in the *bivariate* case, the Geweke measure separately tests for each pair (*x*_*i*_, *x*_*j*_) the cases of *x*_*i*_ → *x*_*j*_ and *x*_*j*_ → *x*_*i*_. In terms of the scenarios described above, the bivariate evaluation of *x*_*i*_ → *x*_*j*_ corresponds to the cases *x*_*j*_ → *x*_*j*_ and {*x*_*i*_, *x*_*j*_} → *x*_*j*_. This means that, when considering 3 or more time series, the Geweke measure would consider only a pairwise analysis.

Similarly, the *conditional*-bivariate implementation of the Geweke measure tests the causal interaction by including in the set of causes of each causality scenario the *M* − 2 time series that are not in the pair under analysis.

In the analysis of the proposed method, we will also consider the subsets of feature space that corresponds to the bivariate and conditional-bivariate cases, by removing scenarios that are not included in those cases. For clarity, we call the two reduced features spaces as *pairwise* (pw) and *conditional-pairwise* (c-pw). In both cases, given *M* time series and selected one as effect, its possible causes define *M* − 1 causality scenarios plus the causality scenario that involves only the effect itself, i.e., *x*_*j*_ → *x*_*j*_. Thus, we obtain *M* causality scenarios for each effect, and *M*^2^ in total. In our example of *M* = 3, the number of scenarios is 9, instead of 21.

### 3.5. Evaluation metrics

In the experiments described in section 4, the ability to identify the correct causal configuration on simulated and real datasets, will be quantified in terms of receiver operating characteristic (ROC) curve and the related area under the curve (AUC). In this way, the obtained results will not be biased by the possible different cost of false discovery that may change in different applications.

The computation of the ROC curve in the cases of the standard Granger causality analysis (GCA, see Barnett and Seth, 2014) and cell-based classification (CBC) is straightforward, because GCA is a conditioned pair-wise method and CBC predicts the single cells of the causality matrix. The ROC curve can then be computed from the false positive (FP) rate and the true positive (TP) rate obtained by varying the classification threshold^5^ and by averaging over all cells and all trials.

In the case of matrix-based classification (MBC), the classification problem is multiclass and the ROC curve cannot be obtained in a straightforward way, in general. Nevertheless, in our specific case, each predicted causal matrix is a binary matrix, as in the case of CBC. The only difference is that, with MBC, all entries of the matrix are jointly predicted instead of being individually predicted each by a different classifier, as in CBC. Anyway, by jointly varying the classification threshold in all entries of the matrix, we can compute the ROC curve for MBC, allowing a fair comparison with CBC and GCA.

## 4. Experiments

The purpose of our empirical analysis is to compare the proposed supervised methods, described in section 3, against the best practice in the literature, which is based on an unsupervised estimate of the parameters of the MAR model. The comparison is performed mainly with synthetic data where the ground truth of effective connectivity is known in advance, by design. Additionally, on real data, we investigate the behavior of the supervised approach when the underlying exact generative model is not known in advance. To conclude, we also report the empirical investigation proposed by the Causal2014 challenge^1^.

### 4.1. Data generation process and feature space

Before describing each experiment, we provide details on the initialization of the MAR model to generate the dataset **L** and on how to create and improve the feature space described in section 3.3. The parameters of the MAR model were set as *p* = 10, *N* = 6, 000, and *M* = 3. Regarding the parameter γ, since the presence of additive noise affects the performance of a Granger-based metric, we generated two versions of the **L** dataset. One version that we call **L**_MAR_, contains only the autoregressive component and no noise corruption. This practically means keeping γ = 0 in Equation (1). In a second version, with explicit noise corruption, γ is picked uniformly at random for each trial. We refer to this last dataset as **L**. Given this setting, there are 2^6^ = 64 possible causal graphs/configurations. One thousand trials were generated for each configuration, thus in total 64,000 trials comprised **L**_MAR_ and **L**.

As explained in section 3.3, as feature space we computed two regression metrics: the mean square error and the coefficient of determination *r*^2^. Both were included because we noticed a significant improvement in the cross-validated score, although, intuitively, they could seem redundant. Additionally, we included an estimate of the Granger causality coefficients^6^. As a further step, we enriched the feature vector by applying standard feature engineering techniques, like simple basis functions. These consisted in extracting the 2nd power, 3rd power, and square root of the previously defined features, together with the pairwise product of all features. Adding extracted features was motivated by the need to overcome the limitation of the adopted linear classifier, see Domingos (2012).

### 4.2. Experiments with the same process of data generation

The experiments presented here have in common that the same process of data generation was used for both the training and the evaluation sets. The experiments with simulated data were designed according to the three main components of the supervised approach: (i) the generative model, (ii) the encoding of the signals into the feature space, and (iii) the use of a classification task.

The first experiment aimed to investigate the effect of including additive noise to the data generation process. Both the unsupervised (GCA) and supervised methods were applied to the two datasets **L**_MAR_ and **L**. For the implementation of GCA, we adopted the toolbox proposed in Barnett and Seth (2014). For the supervised approach, after the mapping of the datasets to the proposed feature space, the logistic regression classifier^7^, with ℓ_2_ regularization, was applied in a five-folds cross-validation framework.

The second experiment aimed to characterize the properties of the feature space proposed in section 3.3, that we call *complete* feature space, and to compare it with its pairwise (pw) and conditional-pairwise (c-pw) versions described in section 3.4. Such restricted/reduced feature spaces were introduced to mimic the Geweke measure, which addresses the bivariate case. The aim is to understand the gain of introducing the *complete* feature space that accounts also for the multivariate case.

The third experiment considered the two alternative schema to formulate the classification task: the matrix-based classification (MBC), which jointly predicts all entries of the causal matrix, and the cell-based classification (CBC), for which each matrix cell refers to a different binary classifier, see section 3.2. Since *M* = 3, in the case of MBC we trained one classifier to predict among 64 different classes, one for each possible causal configuration matrix. In the case of CBC, 6 binary classifiers were trained, one for each cell of the causal matrix. Both versions were applied to the two simulated datasets **L**_MAR_ and **L**.

As an additional evaluation of the supervised approach, here we report the detail of our submission to the Causality2014 challenge. In this setting we know in advance the generative model, i.e., the MAR model, but the ground truth of the causal graph of each trial is unknown. We used the **L** dataset as training set with the MBC method with the complete feature space. The posterior probabilities computed by the logistic regression classifier were converted into predicted classes considering the costs provided by the competition for true positives (+1) and false negatives (−3).

### 4.3. Mismatch between generative processes

In this experiment, we artificially introduced a mismatch between the generative model of the training set and the actual process of signal generation. This is a frequent scenario in practical cases, because generative models are only approximations of the real physical process creating the data. For this reason, we wanted to compare the proposed supervised approach with respect to the standard analysis under such scenario. In practice, we applied CBC to the **L** dataset after training it on the **L**_MAR_ dataset and, as feature space, we adopted its complete version.

As a second experiment on the mismatch of the generative processes, we trained the CBC method on the **L** dataset and tested on the real neural recording dataset described in section 2.2. The experiment was repeated with different configurations, i.e., by changing the sampling frequency of the neural signals and the related model order *p*. As sampling frequency, we set it to 600, 800, and 1,000 Hz and the model order was computed in order to have time windows of 5, 10, 15, 20, and 25 ms. For each pair of sampling frequency and model order the AUC was computed using as true causal graph the causal chain reported in section 2.2, i.e., EC3 → CA1 → EC5, as in van Strien et al. (2009).

## 5. Results

In this section, we report the results of the multiple experiments described in section 4. There, we presented two groups of experiments that we report here too.

In the first group of experiments the model of data generation is exactly the same of the dataset to be analyzed. In other words, the training and testing sets of the supervised approach are generated with the same data generation process. The results of the first experiment, i.e., comparing GCA and the propose supervised methods on data with and without additive noise, are presented in Table 3 as ROC AUC scores (higher is better). As expected, with no additive noise, see row **L**_MAR_, all methods predict identically, because classification is perfectly accurate in all cases. When adding noise, i.e., row **L**, the AUC score changes from 0.72 for GCA to 0.91–0.92 for the supervised methods.

**Table 3:** AUC values of GCA, CBC, and MBC on the two datasets **L**_MAR_ and **L**.

	**GCA**	**CBC**	**MBC**
**L**_MAR_, i.e., γ = 0	1	1	1
**L**, i.e., 0 ≤ γ ≤ 1	0.72	0.92	0.91

*The standard deviation is lower than 0.0015 in all cases*.

The second experiment of the first group compares the different features spaces for the supervised approach. In Table 4, the AUC of the complete feature space (columns CBC), of the pairwise one (column CBC pw), and of the conditional-pairwise one (column CBC c-pw), are reported. The corresponding ROC curves are illustrated in **Figure 4**.

**Table 4:** AUC values of CBC with the complete and reduced feature spaces, on **L**_MAR_ and **L**.

	**CBC**	**CBC c-pw**	**CBC pw**
**L**_MAR_, i.e., γ = 0	1	1	1
**L**, i.e., 0 ≤ γ ≤ 1	0.92	0.91	0.90

*The standard deviation is lower than 0.0015 in all cases*.

**Figure 3:**
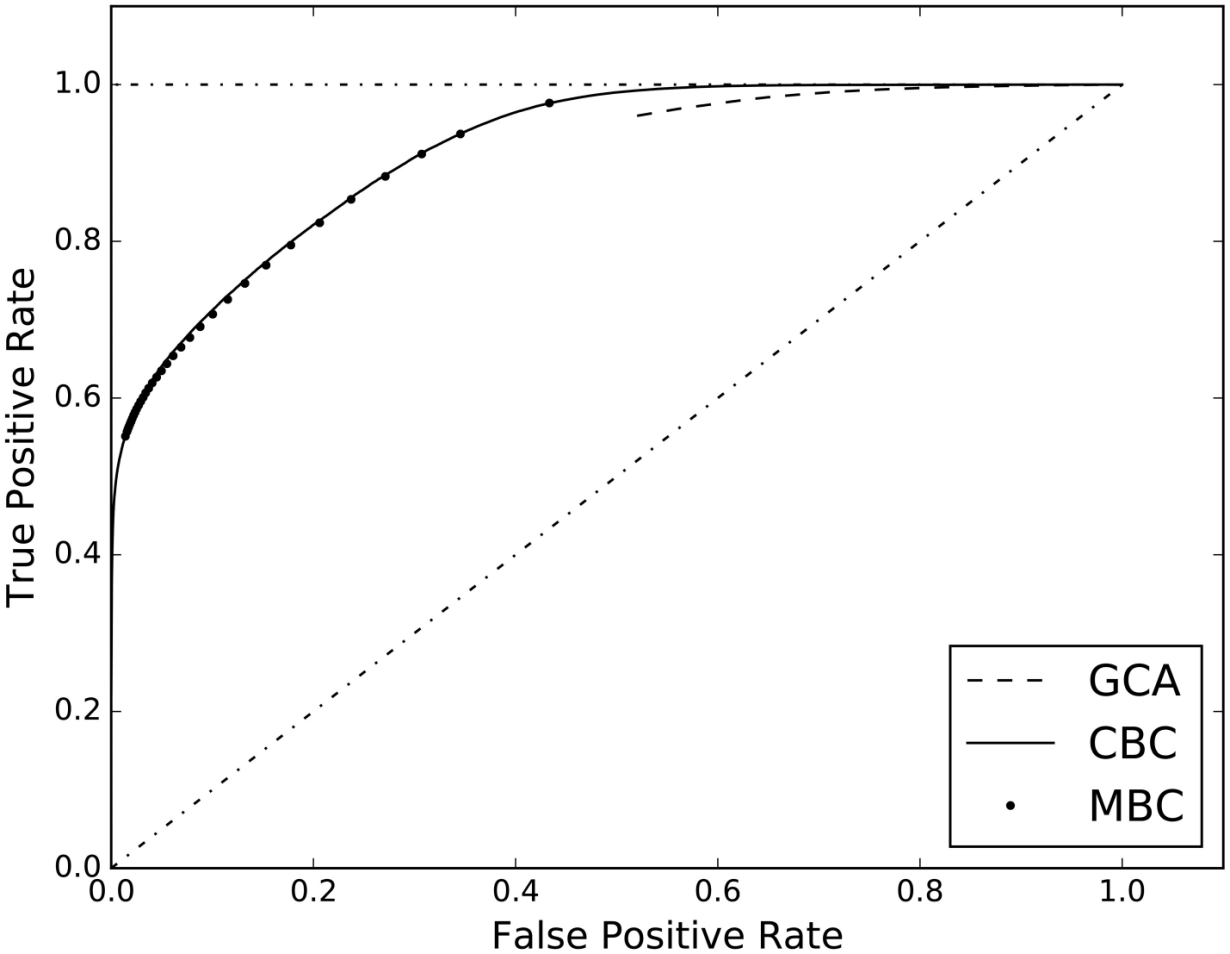
ROC curves estimated on the results of the three analyzed causal inference methods: Granger Causality Analysis (GCA), Cell-based Classification (CBC), and Matrix-based Classification (MBC).

The third experiment of the first group, compares our two different approaches to classification, i.e. the cell-based (CBC) and the matrix-based (MBC) ones. In Table 3, columns 2 and 3, the AUC scores are reported together with those of GCA. The full ROC curve is presented in Figure 3.

The first group of experiments is concluded by the results of the Causal2014 challenge, reported in Table 5. The results are five-fold cross-validated on the training set, because the causality matrices of the test set of the competition were not disclosed. The table reports the confusion matrices of GCA, CBC, and MBC estimated on **L**, following the competition guidelines.

**Table 5:** Confusion matrices of GCA, CBC, and MBC on the Causal2014 dataset, taking into account for the bias for reducing the false-positives.

		**Pred. (GCA)**				**Pred. (CBC)**				**Pred. (MBC)**	
		1	0			1	0			1	0
True	1	99.6%	0.4%	True	1	59.4%	40.6%	True	1	57.8%	42.2%
	0	80.1%	19.9%		0	2.8%	97.2%		0	2.2%	97.8%

*The values are conditional probabilities given the true class, i.e., each row sums up to 1*.

The second group of experiments, investigates the effect of a generative model that differs from the actual generation process of the data to analyze, i.e., there is a mismatch between the two models. The first experiment, where CBC was trained on **L**_MAR_ and tested on **L**, resulted in a ROC AUC score of 0.85. Despite the difference between training set and testing set, the result is superior to the 0.72 obtained by GCA, see Table 3. The results of the second experiments, of CBC on the neural recording dataset (see section 2.2), are reported in Table 6, in terms of AUC score for different choices of the sampling frequency and order of the MAR model (*p*), i.e., the window width. We computed the AUC score also for GCA, obtaining chance-level results, i.e., AUC ≈ 0.5, in all cases. We observed that GCA estimated the existence of causal links in almost all cases/interactions, clearly generating a very large number of false positives. At the same time, we observed that the neural recording data have high autocorrelation and cross-correlation, which may explain such behavior.

**Table 6:** AUC computed by applying CBC to the empirical dataset with different sampling frequencies and time window widths.

	**5 ms**	**10 ms**	**15 ms**	**20 ms**	**25 ms**
600 Hz	0.80	0.82	0.82	0.83	0.82
800 Hz	0.82	0.82	0.82	0.73	0.62
1 kHz	0.82	0.82	0.75	0.61	0.64

*The standard deviation is lower than 0.009 in all cases*.

## 6. Discussion

In this paper, we propose a new approach for causal inference in the framework of machine learning. Specifically, we developed a classification-based method by assuming a model for the stochastic process and a causality measure, and created a suitable feature space. Our idea is to use the model to generate a simulated dataset, representative of the problem of which we want to infer the causal interactions. Then we map this dataset into a suitable feature space. After that, a classifier is trained on the dataset in order to predict the causal graph of a future set of time series, i.e., to predict a set of binary variables. As a consequence, the causal inference is directly dependent both on the chosen generative model and on the designed feature space.

We put this general framework in practice, by customizing it in the case of the Geweke causal inference in time, see section 3, and, as a consequence, of the autoregressive model as the generative process of the multivariate time series. Moreover, another consequence is the assumption of precedence and predictability in time, for the identification of a causal interaction. In sections 3 and 4, we designed a feature space coherent.

In the experiments of section 4, we compared the performance of different methods for causal inference, when applied to a multivariate autoregressive dataset, with and without additive uncorrelated noise. The results are shown in terms of AUC value and ROC curve, see Figure 3. The estimated AUC of each method on each dataset is reported in Tables 3, 4. In the absence of correlated noise, i.e., with dataset *L*_MAR_, all methods perfectly predicted the correct causal configurations, which is a positive sanity check of the supervised approach. With the presence of additive noise, predicting the correct causal configuration becomes more difficult. In particular, we observed that GCA is more sensitive to additive noise than the supervised approaches, scoring AUC = 0.72, with respect to 0.90–0.92 of the supervised methods. Figure 3 confirms that both the supervised methods CBC and MBC perform better than GCA. It is interesting to note that the ROC curve of GCA does not exist for false positive rate lower than 0.55. This occurs because the poor granularity of the scores of GCA does not allow to put thresholds that result in a false positive rate lower than 0.55. Specifically, GCA assigns probability 1.0 to a large amount of causal interactions that are not existent. In these result and other experiments, we observed that GCA tends to overestimate the presence of causal interactions. Differently, both CBC and MBC have much more granularity and higher AUC scores, i.e., 0.92 and 0.91, respectively. Given that both CBC and MBC operate on the same feature space, we can conclude that a joint prediction of all causality interactions, which is what MBC provides, does not result in an advantage over the individual predictions of each interactions, which is what CBC provides.

**Figure 4:**
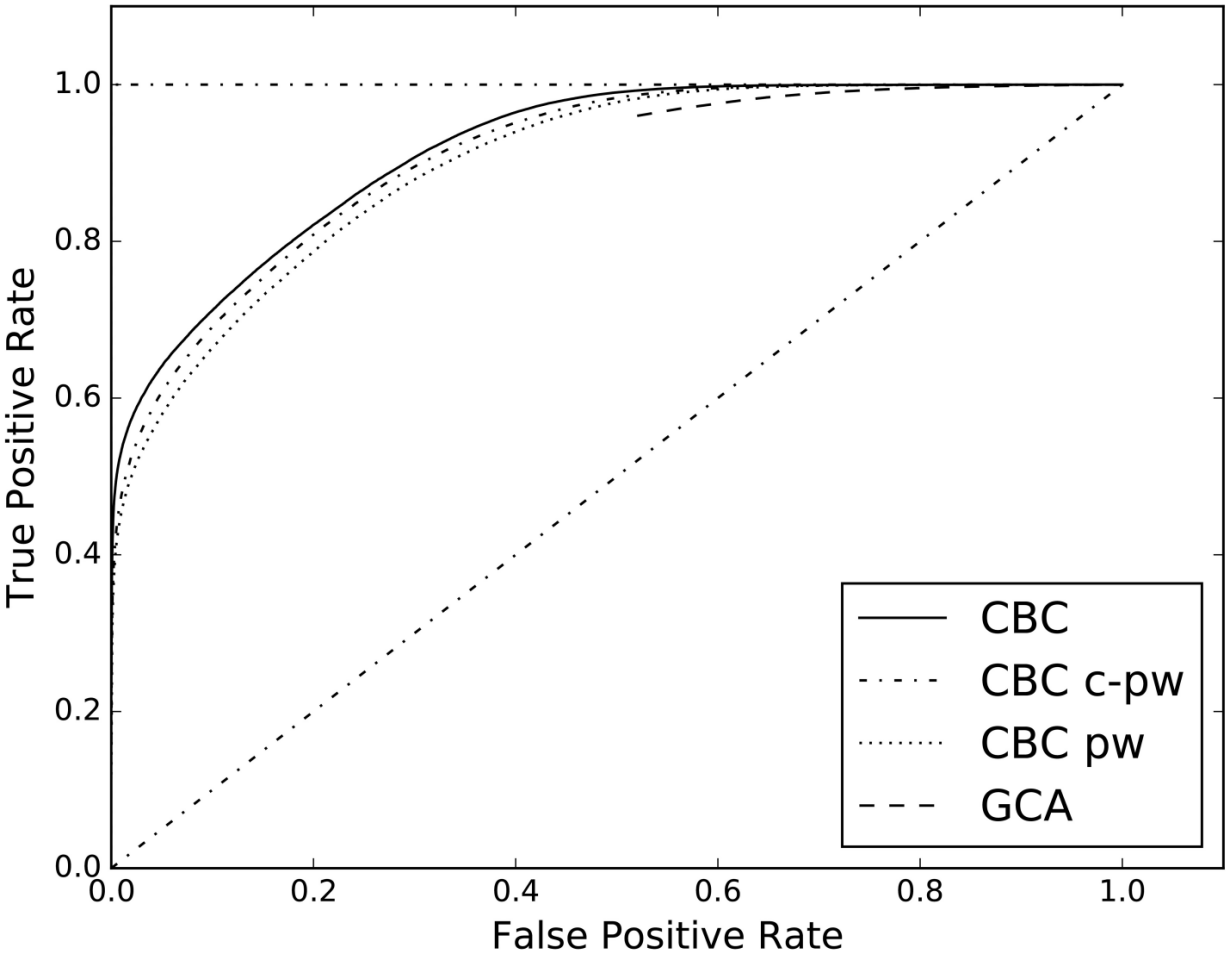
ROC curves estimated on the results of CBC when applied on three different feature spaces: the complete one in contrast with the pw and c-pw ones. The ROC curve of GCA is shown as benchmark.

The proposed supervised approach allows to study multivariate causal interactions. This is different from the Geweke measure, that is a conditioned pairwise method. In the supervised case all the multivariate dependencies among time series are taken into account through the causality scenarios included in the designed feature space, see section 3.3. For this reason, the proposed approach goes beyond what the pairs of cause/effect time series can give. In Figure 4 and Table 4 the results of the analysis on the role of the proposed features space are reported. When considering only the pairwise (CBC pw) and conditional pairwise (CBC c-pw) portions of the feature space, the AUC score is lower than the full feature space (CBC), even tough by a margin.

Considering the specific case of the Causal2014 challenge, we reported in Table 5 the confusion matrices computed with GCA, CBC, and MBC on the training set through cross-validation, considering the cost model defined in the competition, see section 4.2. From this example, we clearly see that GCA provided a very large fraction of false positive, i.e., 80.1%. Differently, both CBC and MBC correctly followed the bias of the competition of reducing the number of false positives, which was 2.8 and 2.2% respectively. Our submission to the competition, with MBC^8^, reached the 2nd place in the ranking, which is positive evidence that, in the case of the Geweke measure, the supervised approach is a meaningful alternative to the current state of the art unsupervised causal inference methods.

In practical cases, generative models may not accurately describe the observed data coming from neuroimaging experiments. For this reason, we wanted to test the effect of introducing a systematic change between the training set and the testing set. Such change may have particularly negative impact for classification-based methods. Then, CBC was trained on *L*_MAR_ and then tested on *L*. As reported in section 5, in this case AUC dropped to 0.85, from 0.92 of the case where *L* was both the training set and the testing set. Such result is still superior to AUC = 0.72, obtained with GCA. Such evidence supports the hypothesis that CBC is also robust to some violations in the assumption of the generative model.

On the neural recordings dataset introduced in section 2.2, the assumption of the MAR model may be incorrect. In section 4, we reported that on such data GCA performed poorly, around chance-level, in all cases. This may be explained by both incorrect assumptions and by the high autocorrelation and cross-correlation in the time series. Differently from GCA, in Table 6 we show that CBC reaches high AUC scores, i.e., around 0.82, for all sampling frequencies. We notice that, for larger time windows and higher frequencies, the AUC drops to 0.61, probably due to the increase in high frequency noise in the data. Nevertheless, it has to be noted that these results assume the validity of the causal chain EC3→CA1→EC5 that was introduced in van Strien et al. (2009).

### 6.1. Computational limitations

In the experiments proposed in this work, we limited the number of time series to *M* = 3. Following the explanations in sections 3.2 and 3.3, this results in 64 classes, in case of MBC, or 6 binary problems, in case of CBC, and a feature space of 21 dimensions^9^. The first and the last number increase exponentially with *M* and the second quadratically with *M*. For *M* = 4, the three numbers become 4,096, 12, and 60, respectively. For this reason, MBC becomes unfit to be used when *M* > 3, because the training set necessary to fit the parameters for a very large number of classes would be unfeasible to obtain and to manage. Nevertheless, even the use of CBC cannot address a large number of time series, because the feature space grows exponentially with *M*.

Nevertheless, it is interesting to note that the feature space proposed in this work is not bound to the generative model considered here, i.e., the MAR model. The causality scenarios defined in section 3.3 are based on the causality measure, i.e., the Geweke measure. This opens interesting avenues for further research, which investigates how the inference based on the same feature space would change when different models of the generative process are used.

## 7. Conclusions and future work

In this work, we presented how the problem of causal inference among time series can be tackled with supervised learning methods. We defined a novel feature space based on the principles of Granger causality and trained a classification algorithm on examples generated from the MAR model. We compared the proposed method with a standard approach in the literature, i.e., GCA, and showed a strong reduction in the false positive rate, together with a sizable improvement in AUC score. The experimental evidence in support to our claims comes from simulation and from the analysis of neural recordings from rats.

In future, we want to address some of the limitations of the proposed method. First, the computational problem of dealing with a larger number of time series, that could be addressed by limiting the number of causality scenarios to a subset of interest for the specific application. A second limitation is the current use of the MAR model. We are already working on alternative generative models, which are known to provide a more accurate representation of the neural recordings. A further interesting future perspective is to extend the method beyond the observational assumption, in order to deal with interventional data, thus embracing other causality frameworks. In this way, issues such as confounding variable could be attacked, leading to a more accurate detection of causality among timeseries.

## Author contributions

DB, EO, and PA: conception and design of the work, critical revision, and final approval for publishing. DB and EO: method implementation and data analysis; DB: article drafting.

### Conflict of interest statement

The authors declare that the research was conducted in the absence of any commercial or financial relationships that could be construed as a potential conflict of interest.

## Footnotes

^1^http://www.biomag2014.org/competition.shtml, see “**Challenge 2**: Causality Challenge.”

^2^Represented as a 3 × 3 binary matrix.

^3^Python implementation at: https://github.com/FBK-NILab/supervised_causality_detection

^4^The diagonal is not relevant since by definition time series are autoregressive.

^5^We assume to use classifiers that produce a classification score, like the probability of having a causal interaction.

^6^See GrangerAnlayzer in NiTime, http://nipy.org/nitime

^7^http://scikit-learn.org

^8^At the time of the competition we had developed only MBC and not CBC.

^9^Without considering the additional feature engineering step described in section 4.1.
